# Secondary diabetes mellitus in acromegaly: Case report and literature review

**DOI:** 10.1097/MD.0000000000039847

**Published:** 2024-09-27

**Authors:** Jinlin Wang, Zaidong Zhang, Yaru Shi, Wentao Wang, Yanli Hu, Zonglan Chen

**Affiliations:** a Department of Clinical Medicine, Jining Medical University, Jining, Shandong, P.R. China; b Department of Hepatobiliary Surgery, Affiliated Hospital of Jining Medical University, Jining, Shandong, P.R. China; c Department of Emergency Medicine, Linyi People’s Hospital, Linyi, Shandong, P.R. China; d Department of Endocrinology and Metabolism, Affiliated Hospital of Jining Medical University, Jining, Shandong, P.R. China.

**Keywords:** acromegaly, pituitary adenoma, radiotherapy, secondary diabetes, surgical treatment

## Abstract

**Rationale:**

Acromegaly, predominantly resulting from a pituitary adenoma, is marked by excessive secretion of growth hormone (GH) and insulin-like growth factor-1 (IGF-1). However, normalization of blood glucose levels posttreatment is rarely achieved. This case study aims to highlight the diagnostic challenges posed by overlapping symptoms of acromegaly and diabetes, emphasizing the importance of precise diagnosis and effective treatment strategies for optimal patient outcomes.

**Patient concerns:**

A 22-year-old male was hospitalized for diabetic ketoacidosis and exhibited classic signs of acromegaly, such as enlarged hands and feet, and distinct facial changes.

**Diagnoses:**

The patient’s diagnosis of acromegaly, attributed to a pituitary adenoma, was confirmed through clinical observations, laboratory findings (notably raised serum GH and IGF-1 levels, and absence of GH suppression after glucose load during an OGTT), and pituitary MRI scans.

**Interventions:**

The patient underwent 2 surgical tumor resections followed by gamma knife radiosurgery (GKRS). After treatment, GH, IGF-1, and blood glucose levels normalized without further need for hypoglycemic intervention.

**Outcomes:**

Posttreatment, the patient achieved stable GH, IGF-1, and blood glucose levels. The hyperglycemia was attributed to the GH-secreting tumor, and its resolution followed the tumor’s removal.

**Lessons:**

This case emphasizes the need for comprehensive assessment in patients with acromegaly to address coexisting diabetic complications. Surgical and radiotherapeutic management of acromegaly can lead to significant metabolic improvements, highlighting the importance of interdisciplinary care in managing these complex cases.

## 1. Introduction

Acromegaly, a rare endocrine disorder typically resulting from a pituitary adenoma, is marked by the continuous overproduction of growth hormone (GH) and insulin-like growth factor-1 (IGF-1). The abnormal elevation in these hormone levels causes not only classic acromegaly symptoms like limb segment overgrowth and facial feature alterations but also a range of severe systemic health issues, encompassing cardiovascular, respiratory, metabolic, musculoskeletal, and neurological complications. Diabetes mellitus, often a complication of acromegaly, arises mainly from insulin resistance and pancreatic β-cell dysfunction induced by elevated levels of GH and IGF-1. Since the initial clinical report of acromegaly in 1567, there has been considerable advancement in understanding this disease. Nevertheless, diagnosing and treating diabetes as a secondary condition to acromegaly continues to be challenging. In acromegaly, secondary diabetes often presents differently than typical diabetes, necessitating heightened vigilance in diagnosis and treatment by clinicians. This study aims to provide a detailed analysis of the clinical features, diagnostic process, and treatment outcomes of diabetes mellitus secondary to acromegaly in a patient with pituitary tumor-induced acromegaly, thereby offering a deeper understanding of such cases. Additionally, it explores the pathogenesis, diagnostic methods, and therapeutic strategies for diabetes mellitus secondary to acromegaly through a literature review and comprehensive analysis of existing studies. We anticipate that this study will yield comprehensive clinical insights into diagnosing and treating diabetes mellitus secondary to acromegaly, serving as a valuable reference for future clinical practice and research.

## 2. Case presentation

A 22-year-old male patient was admitted in November 2019 with complaints of a headache, enlargement of hands and feet over 4 years, and excessive thirst and drinking for the past 2 months. The patient reported the onset of intermittent, tolerable headaches 4 years ago, without obvious triggers. This was associated with progressive enlargement of the hands and feet, an increase in shoe size by 1 to 2 sizes, and a sensation of swelling in both hands. Additional symptoms included widening of the nose, hypertrophy of the lips, anterior protrusion of the lower jaw, and reverse occlusion, without significant thinning of teeth. The patient denied blurring of vision, visual field defects, panic, chest tightness, chest pain, or significant cold intolerance, but noted malaise, frequent colds, and habitual snoring during sleep. Two months ago, the patient developed excessive thirst, polydipsia, and polyuria, with a daily water intake of approximately 6000 mL. This was accompanied by a weight loss of about 13 kg. The patient did not report significant polyphagia but experienced frequent hunger and blurred vision. There was no numbness in the hands and feet and no history of long-term use of glucocorticosteroids, thiazide diuretics, or other medications. The patient had a 1-year history of hypertension, with a maximum recorded blood pressure of 140/86 mm Hg, which was not treated with medication. Personal, marital, and reproductive histories were unremarkable. There was no family history of pituitary tumors. Physical examination revealed a BMI of 28.08 kg/m². Measurements were as follows: bust 100 cm, waist 90 cm, abdomen 91 cm, and hip 107 cm. The patient exhibited acanthosis nigricans on the neck, bilateral axillae, and groin. Physical features included a large face, hypertrophied lips, a widened nose, a slightly protruding mandible, and thick hands and feet. Cardiopulmonary and abdominal examinations showed no obvious abnormalities.

**Figure 1.**
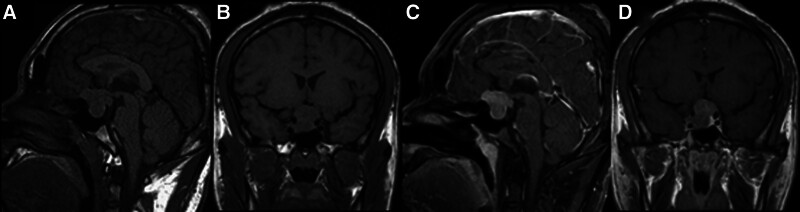
Patient MRI on November 18, 2019 (A and B: T1WI; C and D: enhanced MRI).

After admission, growth hormone (GH) levels were measured at 214.080 ng/mL and insulin-like growth factor-1 (IGF-1) at 951 ng/mL. Pituitary MRI, including both plain and enhanced scans (Fig. 1), revealed a lesion in the sella turcica region, suggesting a high likelihood of a pituitary tumor with dimensions approximately 2.4 cm × 2.7 cm × 1.9 cm. Assessment of anterior pituitary function showed the following: Pituitary-adrenal axis: Cortisol and ACTH secretion levels and rhythms were approximately normal, with a 24-hour urinary free cortisol of 898.0 µg/24h. Pituitary-gonadal axis: Progesterone (P) was 0.37 ng/mL, testosterone (T) was 0.34 ng/mL, estradiol (E2) was <11.80 pg/mL, prolactin (PRL) was 10.65 ng/mL, luteinizing hormone (LH) was 0.93 mIU/mL, and follicle-stimulating hormone (FSH) was 0.63 mIU/mL. Pituitary-Thyroid Axis: Metabolic tests showed FT3 at 3.05 pmol/L, FT4 at 12.80 pmol/L, and TSH at 0.09 mIU/L. Posterior Pituitary Function Evaluation: Plasma osmolality was 281 mOsm/kg H_2_O, and urinary osmolality was 355 mOsm/kg H_2_O. Evaluation of Related Complications: Glucose Metabolism: Fasting blood glucose was 17.7 mmol/L, and glycosylated hemoglobin was 14.3%. Lipid and bone metabolism indices were normal. Abdominal Ultrasound: Revealed a hyperechoic area in the left lobe of the liver; hemangioma could not be excluded. Chest Ultrasound: Also showed a hyperechoic area in the left lobe of the liver; hemangioma was not excluded. Lower Extremity Arterial Ultrasound: No significant abnormalities were observed. Cardiac Ultrasound: At rest, no significant abnormalities in intracardiac structures or blood flow; ejection fraction was 60%. Colonoscopy: No notable abnormalities in the colorectal mucosa. Thyroid and Lymph Node Ultrasound: No definite abnormalities were detected. Visual Fields: Generally normal. Fundus Examination: No obvious retinal hemorrhage or exudation; an area of degeneration was noted in the retina above the left eye. Ophthalmologic Consultation: Diagnosed with retinal degeneration in the left eye and high myopia in both eyes. The patient’s serum GH and IGF-1 levels were significantly elevated, and the clinical manifestations were consistent with acromegaly. Combined with pituitary magnetic resonance imaging, the patient was diagnosed with acromegaly, a pituitary tumor, and diabetes mellitus.

**Figure 2.**
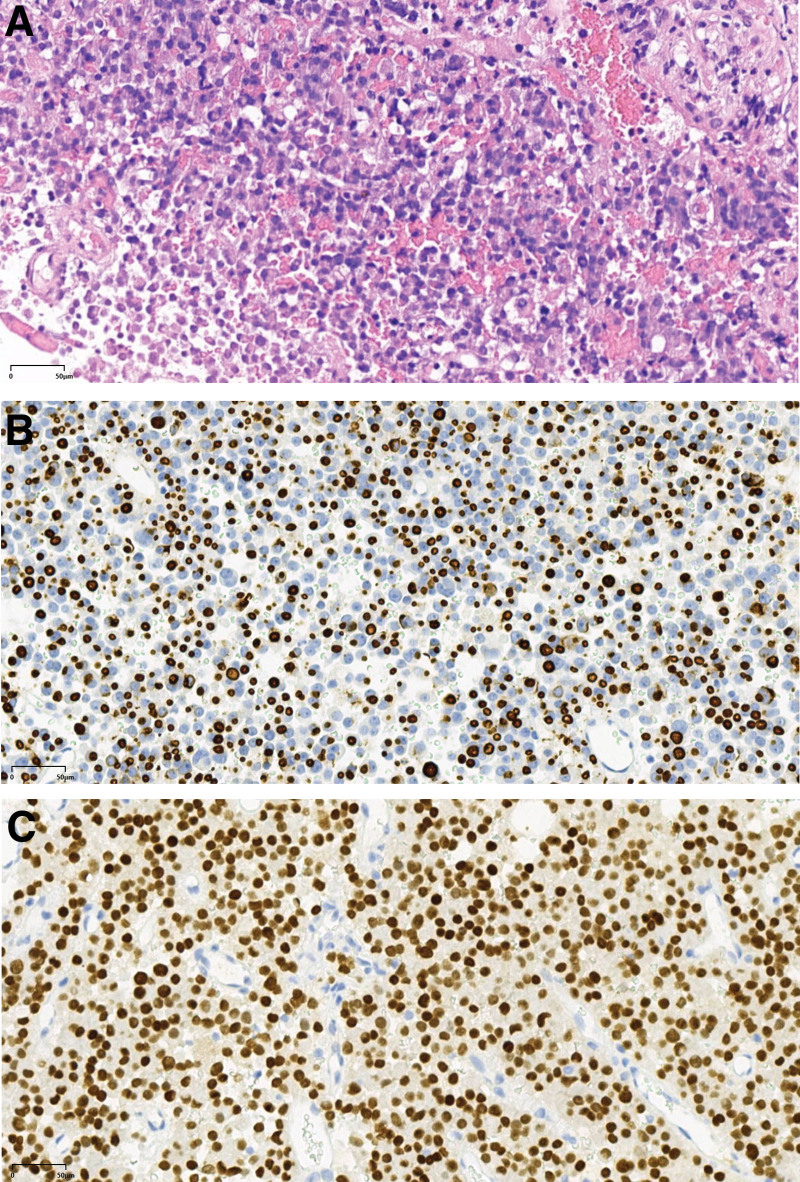
(A) HE staining at 200× magnification revealed tumor cells arranged in sheets. The cells exhibited a smoky to weakly eosinophilic appearance, with marked cellular pleomorphism and eccentrically positioned nuclei. (B) Immunohistochemistry results: CK8/18 Paranuclear fibrosome+. (C) : Immunohistochemistry results: PIT-1+.

After achieving stable glycemic control, the patient underwent their first neuroendoscopic transnasal-pterygoid sinus resection of pituitary lesions on November 28, 2019. Postoperative pathological findings (Fig. 2) confirmed a pituitary adenoma in the sella region. The clinical and immunohistochemical analysis was consistent with a sparsely granulated GH cell adenoma. Immunohistochemistry results were as follows: ACTH(−), FSH(−), GH(+), LH(−), PRL (focally +), TSH(−), CK8/18 (paranuclear fibrosome +), ER(−), P53(−), Pit-1(+), T-pit(−), SF-1(±), and Ki-67(+, approximately 3%). After the operation, the patient was rechecked regularly in our outpatient clinic. In the postoperative period, the patient’s serum GH was 40.343 ng/mL and serum IGF-1 was 1142 ng/mL; in the postoperative period, the patient’s serum GH was 59.360 ng/mL and serum IGF-1 was 1123 ng/mL; These findings suggest that the patient has residual pituitary tumor tissue and has not achieved biochemical remission.

**Figure 3.**
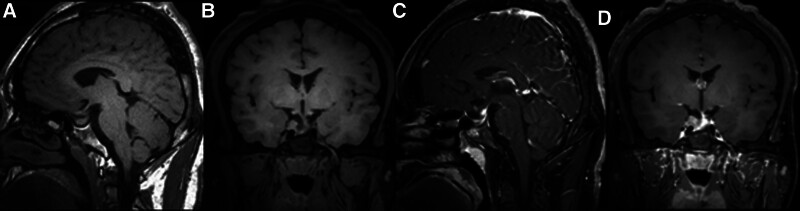
Patient MRI on July 4, 2020 (A and B:T1WI; C and D: enhanced MRI).

On May 18, 2020, the patient underwent a second resection in the sella region. Postoperatively, the patient experienced transient thirst, polydipsia, and polyuria, which worsened but were managed with short-term desmopressin acetate treatment. There was no loss of visual acuity, no visual field defects, no cerebrospinal fluid leakage, and only occasional headaches. After surgery, insulin was discontinued, and the patient switched to saxagliptin 5 mg/d and metformin 500 mg twice daily for glycemic control, achieving target blood glucose levels. The patient was reexamined 2 months after the second surgery with a pituitary MRI (plain and dynamic enhancement, Fig. 3). Serum GH was 5.522 ng/mL and IGF-1 was 805 ng/mL. A 75g OGTT with GH suppression test revealed a serum GH trough of 4.348 ng/mL (Table 1), indicating that biochemical remission had not been achieved after the second surgery. Pituitary function tests showed the following gonadal hormone levels: P 0.56 ng/mL, T 0.59 ng/mL, E2 27.60 pg/mL, PRL 6.29 ng/mL, LH 1.95 mIU/mL, FSH 1.62 mIU/mL. Cortisol and ACTH levels and rhythms, as well as thyroid function, were approximately normal.

**Table 1:** Oral glucose tolerance test for GH suppression – July 4, 2020.

Time (min)	0	30	60	90	120	180
Blood glucose (mmol/l)	5.2	6.1	8.5	–	9.6	6.5
C-peptide (ng/mL)	5.33	9.24	10	–	15.9	21.7
GH (ng/mL)	5.093	5.04	5.859	4.829	4.348	–

**Figure 4.**
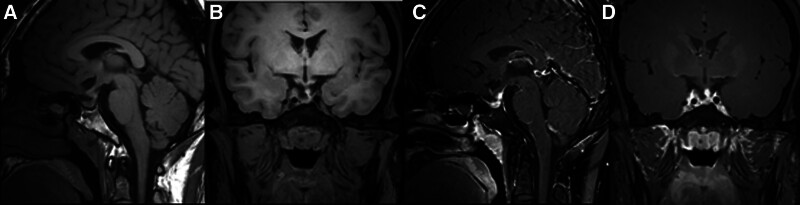
Patient MRI on June 27, 2021 (A and B: T1WI; C and D: enhanced MRI).

Due to the lack of biochemical remission, the patient underwent Gamma Knife treatment on November 15, 2020. Seven months after radiotherapy, a follow-up pituitary MRI (plain scan and dynamic enhancement, Fig. 4) was performed. The patient’s serum GH was 0.782 ng/mL, IGF-1 was 470 ng/mL, and a 75 g OGTT with GH suppression test, as shown in Table 2, was conducted. Fasting blood glucose was 5.6 mmol/L, and glycosylated hemoglobin was 5.5%. Pituitary Function Assessment: Gonadal hormone levels were as follows: E2 12.36 pg/mL, T 0.17 ng/mL, P 0.25 ng/mL, PRL 42.31 ng/mL, LH 0.47 mIU/mL, FSH 1.00 mIU/mL. Cortisol and ACTH rhythms, as well as thyroid function, were approximately normal.

**Table 2:** Oral glucose tolerance test for GH suppression – June 29, 2021.

Time (min)	0	30	60	90	120	180
Blood glucose (mmol/l)	4.4	7.7	7.5	7.2	5.3	4.9
C-peptide (ng/mL)	5.28	14.4	11.1	–	11.4	7.95
GH (ng/mL)	0.87	0.762	0.697	0.623	0.615	0.697

**Figure 5.**
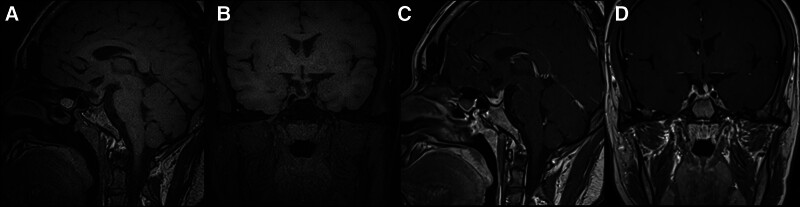
Patient MRI on October 22, 2023 (A and B:T1WI; C and D: enhanced MRI).

In summary, the patient with acromegaly was able to discontinue hypoglycemic medications and achieved satisfactory glycemic control after serum GH and IGF-1 levels normalized following secondary surgery and radiation therapy. This suggests that the patient’s previous hyperglycemia was likely secondary to diabetes mellitus caused by the GH-secreting tumor. In the third year after radiotherapy, IGF-1 was 162.3 ng/mL and GH was 0.61 ng/mL on recheck on October 16, 2023. A follow-up pituitary MRI with plain scanning and dynamic enhancement was performed on October 22, 2023 (Fig. 5). GH was 0.313 ng/mL, IGF-1 was 187 ng/mL, and IGFBP-3 was 6.27 μg/mL. Gonadal Function: P 0.16 ng/mL, T < 0.07 ng/mL, E2 14.95 pg/mL, PRL 35.39 ng/mL, LH 0.27 mIU/mL, FSH 1.57 mIU/mL. Thyroid Function (AF3): T3 4.11 pmol/L, T4 9.60 pmol/L, TSH 1.08 mIU/L. Cortisol and ACTH rhythms were approximately normal.

## 3. Discussion

### 3.1. Etiology and mechanisms

Acromegaly is most often the result of monoclonal expansion of GH-secreting cells within a pituitary adenoma, leading to chronic overproduction of GH and IGF-1. This hormonal imbalance causes numerous systemic complications, encompassing cardiovascular, respiratory, metabolic, musculoskeletal, neurological, and neoplastic issues.^[1]^ The initial clinical case of acromegaly was documented in 1567, with the term “acromegaly” being coined by French neurologist Pierre Marie in 1886.^[2]^ About 99% of acromegaly cases stem from GH-secreting pituitary adenomas. In contrast, pituitary adenomas secreting GH-releasing hormone and ectopic tumors producing growth hormone are uncommon, constituting roughly 1% of cases.^[3]^ Recent research indicates that the molecular biology of sporadic GH adenomas likely involves genes including GNAS, PTTG, PDE4D, and GPR101.^[4–6]^ GNAS activation and PDE4D overexpression can result in cAMP buildup and excessive GH production. Up-regulation of the GPR101 gene, due to Xq26.3 microrepeats on the X chromosome, leads to a less common X-linked acromegaly variant. Additionally, overexpressed PTTG, a protein crucial for chromosomal stability, might contribute to pituitary adenoma formation.

### 3.2. Clinical manifestations and diagnosis

Acromegaly resulting from GH adenoma typically presents with GH overproduction symptoms, tumor compression effects, and multisystem involvement, including respiratory, circulatory, digestive, and skeletal systems. In this case, the patient exhibited classic external acromegaly signs: lip hypertrophy, nose widening, jaw protrusion, and coarse hands and feet. Upon hospital admission, the patient underwent comprehensive multisystem examinations: cardiac, abdominal, and lower limb arterial ultrasounds; thyroid gland and lymph node ultrasounds; chest X-ray; gastrointestinaloscopy; limb nerve conduction velocity tests; visual field; and retinal examinations, which identified peripheral nerve involvement and hypoplasia. The 2020 Chinese expert consensus on acromegaly states that its qualitative diagnosis depends on measuring serum GH and IGF-1 levels and conducting an OGTT GH suppression test. Locoregional diagnosis is based on pituitary magnetic resonance imaging. A multidisciplinary approach is recommended for formulating the optimal treatment plan.^[2]^ Owing to advancements in ultrasensitive GH assays, some experts propose adjusting the GH threshold for diagnosing acromegaly via the OGTT to 0.4 ng/mL, contingent on the assay utilized.^[7]^ In this instance, the patient did not undergo an ultrasensitive GH test. Elevated serum GH and IGF-1 levels were observed, and the OGTT GH suppression test indicated GH levels > 1 ng/mL. These findings, along with the results of pituitary magnetic resonance imaging, confirmed an acromegaly diagnosis attributable to pituitary adenoma.

### 3.3. Mechanisms of secondary diabetes

Numerous studies, both domestic and international, have documented instances of diabetes mellitus coexisting with acromegaly. However, reports of blood glucose normalization postsurgery are comparatively infrequent.^[8–13]^ The primary pathophysiological processes through which acromegaly contributes to diabetes mellitus involve heightened lipolysis and modified fat distribution, leading to increased insulin resistance.^[9]^ The pivotal factor is the mutual antagonism in insulin action by GH and IGF-1: GH promotes insulin resistance, while IGF-1 increases insulin sensitivity. GH may contribute to insulin resistance by boosting free fatty acid (FFA) production, which inhibits glucose transporter proteins in adipose tissue cells.^[14]^ IGF-1 and insulin have a reciprocal relationship: IGF-1 boosts insulin sensitivity and secretion, whereas insulin promotes IGF-1 secretion by augmenting hepatic GH receptor (GHR) responsiveness. Research indicates that when levels of IGF-I, insulin, and S-Klotho rise beyond a certain threshold, IGF-IR may develop relative resistance to IGF-I. This could account for the predominance of GH diabetogenic effects over IGF-1 insulin-sensitizing effects in acromegaly patients.^[15]^

### 3.4. Treatment

The current consensus is that surgical resection remains the preferred treatment for pituitary adenomas, particularly effective for pituitary microadenomas. Pharmacological treatment is primarily indicated for patients unsuitable for surgery or in cases where surgery does not achieve resolution, while radiotherapy is typically reserved as a third-line treatment.^[16]^ Pharmacological options fall into 3 main categories: somatostatin receptor ligands, dopamine receptor agonists (DAs), and GH receptor antagonists. The first generation of somatostatin receptor ligands, including octreotide and lanreotide, targets both SSTR2 and SSTR5 subtypes, demonstrating promising efficacy and safety. Pasireotide, a second-generation SRL, has a broader SSTR affinity, targeting SSTR1, 2, 3, and 5, but its safety profile is less favorable compared to the first generation.^[17]^ The most reliable predictor of SRL efficacy is the expression level of SST2, with lower disease control likely in patients with low expression of this biomarker. Additionally, other factors such as tumor signal intensity on MRI T2-weighted sequences, pretreatment IGF-I levels, age, and body weight may also have predictive value for response to SRL treatment.^[18,19]^ The 2020 Expert Consensus on Acromegaly Multidisciplinary Management suggests considering reoperation for patients with suboptimal surgical results and considerable residual tumor, or for those whose initial surgery was unsuccessful and have potentially resectable residual tumor.^[16]^ In a retrospective study of 522 acromegaly patients, examining the correlation between diabetes mellitus and hypertension prevalence with acromegaly biochemical markers, it was discovered that diabetes mellitus prevalence was reduced in patients who achieved biochemical remission postsurgery, as opposed to those with active acromegaly.^[20]^ This study underscores the necessity of a multifactorial diagnostic and treatment strategy for acromegaly and diabetes mellitus to mitigate misdiagnosis and treatment delays. Factors such as gender, tumor volume, suprasellar extension, prior radiotherapy history, average radiation dose to the pituitary gland, isodose line, borderline dose, and maximum dose have been reported as potential contributors to the development of hypopituitarism in patients with pituitary adenomas following their first Gamma Knife Radiosurgery (GKRS) treatment.^[21]^ In a multicenter international study, treatment with lower isodose lines was significantly associated with an increased incidence of new-onset hypopituitarism (*P* = .001, HR = 1.38). Specifically, a 10% reduction in the isodose line was linked to a 27% increase in the risk of developing hypopituitarism after GKRS. The study also found that previous surgical history, preexisting hypopituitarism, borderline and maximum dose, cavernous sinus invasion, suprasellar extension, and treatment volume were not significantly associated with the onset of hypopituitarism following GKRS.^[22]^ However, Jonathan Pomeraniec et al demonstrated that nonfunctioning adenomas, younger age, higher doses to the limbic system, and increased radiation exposure to the pituitary stalk and normal pituitary gland were independent predictors of new-onset or exacerbated hypopituitarism.^[23]^ Meanwhile, Yu Jinxiu et al identified large tumor size (≥5 cm³) and tumor progression after GKRS as risk factors for new-onset hypopituitarism following a single GKRS treatment. Consequently, regular endocrine follow-up is essential.^[21]^

**Figure 6.**
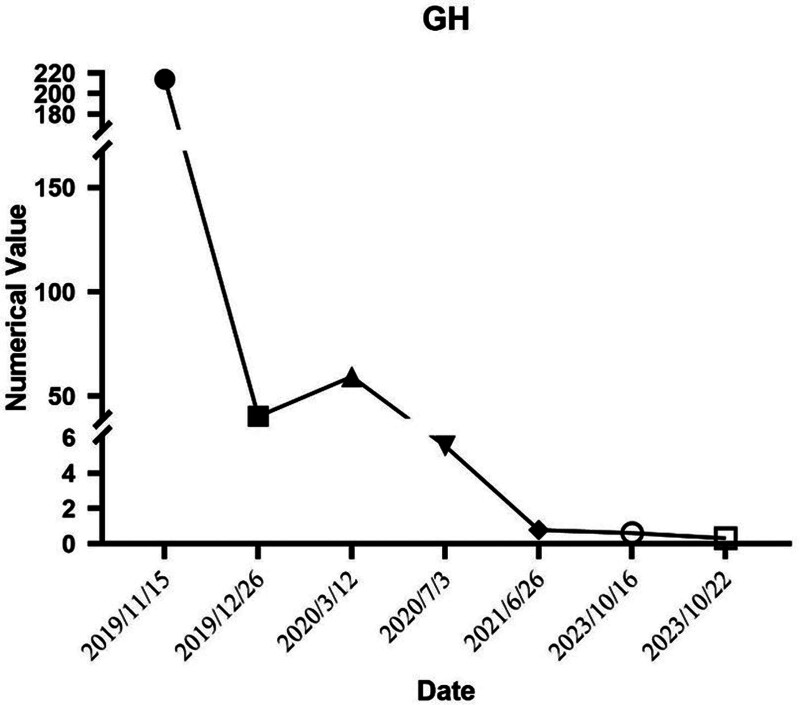
Serum GH level changes.

**Figure 7.**
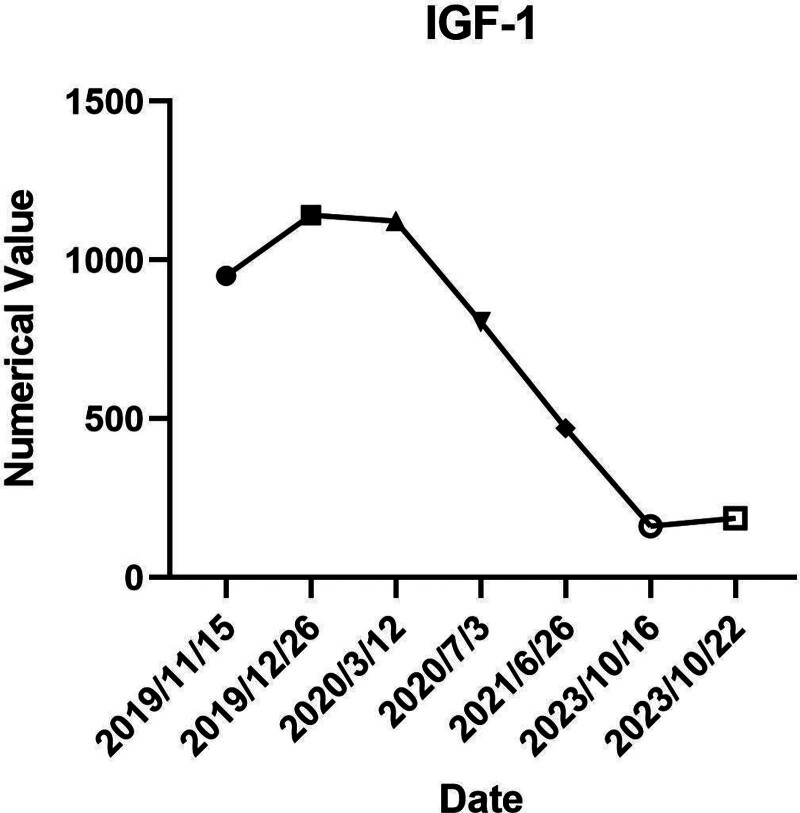
Serum IGF-1 level changes.

The initial symptom of acromegaly in this patient was diabetic ketoacidosis. Upon admission, elevated GH and IGF-1 levels were noted. Postnasal-pituitary sinus lesion resection, biochemical parameters showed improvement. Subsequent outpatient monitoring revealed increased GH and IGF-1 levels, prompting a second surgery due to suspected pituitary tumor recurrence. Postoperatively, GH and IGF-1 levels decreased, yet remained high, and GH suppression was insufficient post-OGTT. Consequently, the patient received gamma knife radiotherapy as a third intervention. Following 2 surgeries and gamma knife therapy, the patient’s GH, IGF-1, and blood glucose normalized, eliminating the need for glucose-lowering drugs. Previously high blood glucose was attributed to the GH tumor. For detailed GH and IGF-1 changes (Figs. 6 and 7). Following a thorough evaluation, the patient’s pituitary GH tumor was managed with combined surgical and radiotherapeutic approaches. This resulted in enhanced GH and IGF-1 control and normalization of blood glucose levels, fulfilling biochemical remission criteria. Additional hormonal axis evaluations indicated normal blood cortisol, ACTH secretion, and biorhythms, with sex hormone levels pointing to hypogonadotropic hypogonadism. The patient’s symptoms, including erectile dysfunction, reduced libido, and sparse axillary hair, suggested concomitant hypothalamic-pituitary-gonadal and thyroid axis hypogonadism, without adrenal axis impairment. Currently, the patient is receiving testosterone undecanoate and levothyroxine therapy, with ongoing regular monitoring and dynamic observation planned.

## 4. Conclusion

In summary, overproduction of GH and IGF-1 due to pituitary adenoma not only causes distinctive cosmetic alterations but also results in multisystem health issues. Although surgical resection is the preferred treatment, the development of pharmacological and radiological therapies provides additional patient options. Moreover, the infrequent normalization of blood glucose postsurgery in diabetes mellitus secondary to acromegaly highlights the need for more thorough consideration of underlying causes in the diagnosis and treatment of diabetes mellitus. Diabetes mellitus represents a complex, multifactorial syndrome rather than a singular disease. In certain cases, acromegaly can obscure or replicate diabetes mellitus symptoms, potentially leading to misdiagnosis or treatment delays.

## Limitations of the Study

This study has several limitations. Firstly, it is based on a single case, which limits the generalizability of the findings. Secondly, the absence of long-term follow-up prevents us from assessing the durability of hormone and blood glucose normalization or the risk of symptom recurrence. Additionally, the study lacked a control group and did not compare different treatment methods, which restricts our ability to evaluate the relative effectiveness of the interventions.

## Author contributions

**Conceptualization:** Zonglan Chen.

**Funding acquisition:** Zonglan Chen.

**Writing – original draft:** Jinlin Wang.

**Writing – review & editing:** Zaidong Zhang, Yaru Shi, Wentao Wang, Yanli Hu, Zonglan Chen.
